# Simultaneous Multifocal Plane Fourier Ptychographic Microscopy Utilizing a Standard RGB Camera

**DOI:** 10.3390/s24144426

**Published:** 2024-07-09

**Authors:** Giseok Oh, Hyun Choi

**Affiliations:** Department of Mechanical Convergence Engineering, Gyeongsang National University, 54 Charyong-ro 48beon-gil, Uichang-gu, Changwon 51391, Republic of Korea; ogs9639@gnu.ac.kr

**Keywords:** Fourier ptychography microscopy, computational imaging, multifocal plane imaging, RGB channel split

## Abstract

Fourier ptychographic microscopy (FPM) is a computational imaging technology that can acquire high-resolution large-area images for applications ranging from biology to microelectronics. In this study, we utilize multifocal plane imaging to enhance the existing FPM technology. Using an RGB light emitting diode (LED) array to illuminate the sample, raw images are captured using a color camera. Then, exploiting the basic optical principle of wavelength-dependent focal length variation, three focal plane images are extracted from the raw image through simple R, G, and B channel separation. Herein, a single aspherical lens with a numerical aperture (NA) of 0.15 was used as the objective lens, and the illumination NA used for FPM image reconstruction was 0.08. Therefore, simultaneous multifocal plane FPM with a synthetic NA of 0.23 was achieved. The multifocal imaging performance of the enhanced FPM system was then evaluated by inspecting a transparent organic light-emitting diode (OLED) sample. The FPM system was able to simultaneously inspect the individual OLED pixels as well as the surface of the encapsulating glass substrate by separating R, G, and B channel images from the raw image, which was taken in one shot.

## 1. Introduction

Fourier ptychographic microscopy (FPM) is an imaging technology that overcomes the limitations arising from the space–bandwidth product of conventional microscopy, enabling a wide field of view (FOV) at high resolution [1,2,3,4]. FPM can easily acquire high-resolution images by reconstructing several images obtained using light sources that illuminate the sample at various angles in the Fourier plane. Consequently, due to its wide FOV and high resolution, FPM is widely applied in various fields, such as biomedical research [5,6] and industrial instrumentation [7].

FPM achieves high-resolution images by exploiting the wide FOV of lenses with low NAs. However, due to this wide FOV, sample tilt increases the probability of deviating from the depth of field of the objective lens, introducing defocus aberration. Therefore, effective strategies are required to compensate for the generated defocus in FPM. The embedded pupil function recovery (EPRY) algorithm can effectively correct defocus while performing image reconstruction [8]. Fast digital refocusing can extend the depth of field by utilizing lateral image shifts caused by angle-varied illuminations [9]. However, while the EPRY algorithm and fast digital refocusing algorithm can easily correct defocus using post-processing, they cannot handle large aberrations. 

In a previous study, we proposed a refocusing method that could adjust the focus without lens movement using conjugate mirror scanning [10]. This method allows a large DOF but requires additional hardware and mechanical movements. 

In this study, we propose an FPM system that can image three multifocal planes simultaneously using a color camera. The light-emitting diode (LED) arrays used in FPM can emit three wavelengths. Then, using a single material aspherical lens, these three wavelengths are focused on different focal planes. This multifocal plane image is acquired as a color camera, and a high-resolution reconstruction process is applied. With this approach, the DOF can be extended by observing the multifocal plane without physical movement. Multifocal plane microscopy can concurrently image different focal planes within a sample. It overcomes depth identification challenges encountered by existing single-plane microscopes and can determine the location and structure of the sample along the z-axis [11]. Previously proposed methods for multifocal plane imaging required separate optical components, such as an off-axis Fresnel zone plate [12], or required the use of volume holograms [13]. However, our proposed FPM can obtain simultaneous multi-focus images using only one camera, as the color camera detects three wavelengths separately. Additionally, a method similar to the one proposed in this study expands the DOF of a barcode decoder by providing different responses to RGB channels using a phase mask [14]. However, when the DOF is expanded using RGB, high-resolution imaging is not possible. The simultaneous multifocal plane FPM we developed in this study uses only a one-color camera, has a very simple optical structure, and implements a very efficient FPM reconstruction algorithm to achieve high-resolution multifocal images.

## 2. Methods

### 2.1. Formation of Wavelength-Dependent Multifocal Planes

When a ray of light passes through a lens, a focus is formed according to the Lensmaker’s equation [15]:

(1) $$\frac{1}{f}=\left(n-1\right)\left(\frac{1}{R_{1}}-\frac{1}{R_{2}}\right)$$

where f is the distance to the focal plane of the lens, *n* is the refractive index of the lens, and $R_{1}$ and $R_{2}$ are the radii of curvature of the two lens surfaces. When the wavelength of the optical field entering the lens changes, the refractive index of the lens, *n*, changes. Therefore, as the wavelength of the optical field becomes shorter, the refractive index n becomes larger, and the distance to the focal plane decreases. Generally, a wide-spectrum light source introduces chromatic aberrations that negatively affect image quality. Consequently, the lens must be designed to correct these chromatic aberrations.

**Figure 1:**
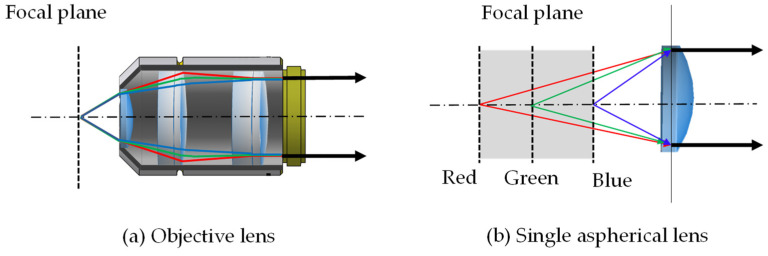
(**a**) Ray tracing of the objective lens. Even if the wavelength of the lighting used varies, the light is focused on a single focal plane. (**b**) Ray tracing of a single aspherical lens. Focal planes at different positions are formed depending on the wavelength of the lighting used.

Figure 1a shows the ray tracing of the general objective lens. In general, objective lenses, as mentioned above, lens groups of various materials are used to correct changes in the focal plane according to wavelengths to prevent chromatic aberration. Figure 1b shows the ray tracing of a single aspherical lens. When only a single material lens is used, the focal plane changes significantly depending on the wavelength change compared to a general objective lens. In this study, a single diffraction-limited N-BK7 aspheric lens (AL2550G-A, Thorlabs, Newton, NJ, USA) was utilized as the objective lens to maximize the change in focal length due to the change in wavelength. The lens properties are summarized in Table 1. The design wavelength indicated by the manufacturer is 532 nm, and the effective focal length (EFL) is 50 mm. In the experimental setup, the FPM LED array emits an optical field with wavelengths of 627 nm (R), 520 nm (G), and 464 nm (B), and the corresponding EFL is 50.409 mm, 49.945 mm, and 49.559 mm, respectively. Therefore, in this FPM system with a single aspherical lens, the focal plane spacings that can be obtained by changing the LED wavelength are 0.464 mm (between R and G focal planes) and 0.386 mm (between G and B focal planes).

### 2.2. Acquiring Multifocal Plane Images Simultaneously Using a Color Camera

The channel splitting algorithm is easily integrated into FPM’s image reconstruction algorithm, and one image capture can obtain three depth images. First, all RGB LEDs are turned on to produce white light illumination. Then, the image is captured using the color camera. Finally, the RGB channels of the captured color image are separated to obtain images from three multifocal planes. 

**Figure 2:**
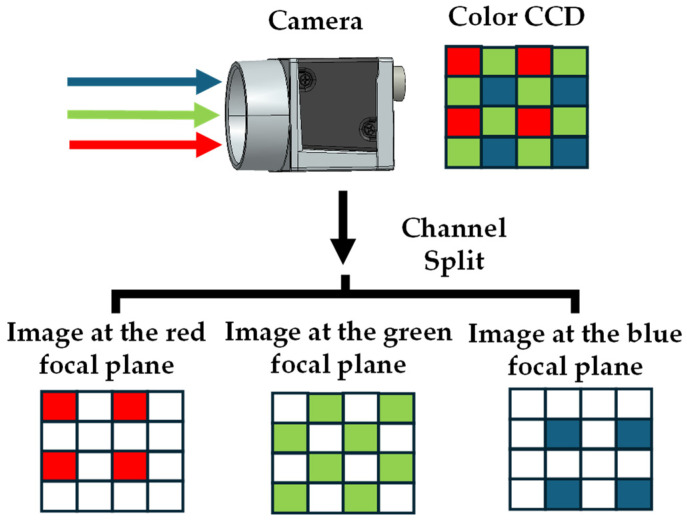
Color camera RGB Bayer pattern. Extracted into R, G, and B focal plane images through the RGB channel division algorithm.

The RGB color pixels of the color camera (acA3800-14uc, Basler, Ahrensburg, Germany) are arranged in a Bayer pattern, as shown in Figure 2. The RGB channel splitting algorithm extracts the images captured by each color channel into R, G, and B focal plane images. The main specifications of the acA3800-14um camera are summarized in Table 2.

**Figure 3:**
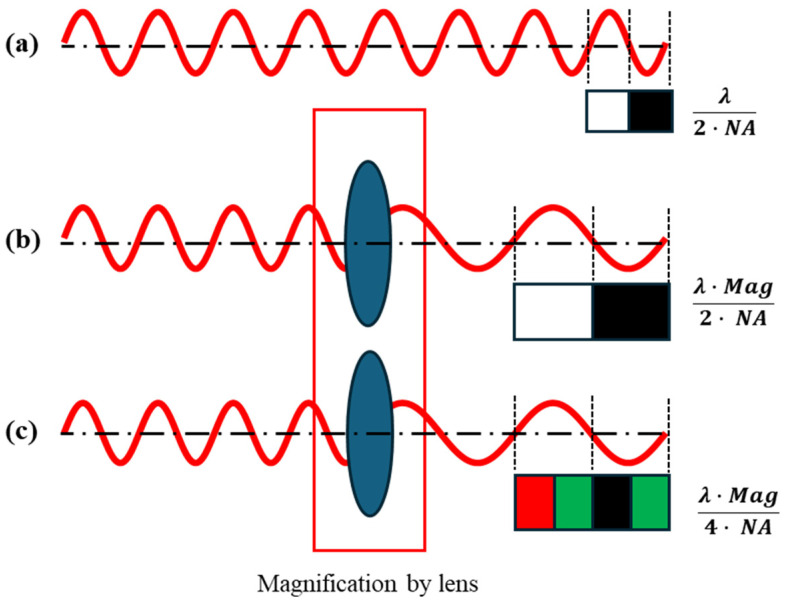
To avoid aliasing, the pixel size must be set to sample one-half of the spatial frequency at the highest resolution (Nyquist criteria). (**a**) The pixel size is expressed as λ/2∙NA. The red line outlines the spatial frequency with the highest resolution in the image, and the pair of white and black squares shows the size of the pixel. (**b**) Considering the magnification of the lens, the minimum pixel size is expressed as λ∙Mag/2∙NA, where Mag is the magnification of the lens. (**c**) For a color camera with a Bayer pattern, the final minimum pixel size is expressed as λ∙Mag/4∙NA. This is because the pixels corresponding to each color pixel are one space apart. If a red optical field is incident on a color camera with a Bayer pattern, light does not enter the pixel of the green color filter, and light only enters the red color filter pixel one space away.

According to the Nyquist theorem, the pixel size must be smaller than λ/(2∙NA). Here, λ is the wavelength of the optical field, and NA is the NA of the objective lens. Pixel sizes larger than the Nyquist limit can lead to pixel aliasing problems in the Fourier domain; therefore, the pixel size of the camera used in FPM should be carefully selected to meet the Nyquist limit. The pixel size, considering the Nyquist criteria, was selected as shown in Figure 3 [16]. 

**Figure 4:**
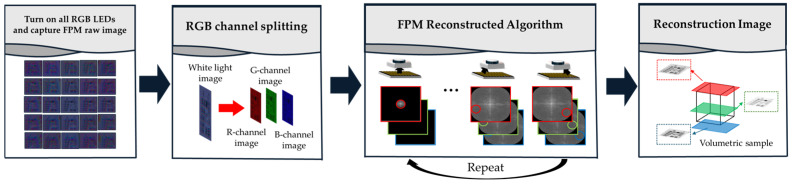
Schematic of simultaneous multifocal plane FPM workflow.

Figure 4 presents the simultaneous multifocal plane FPM workflow. All RGB LEDs are turned on to illuminate the sample so that a raw image with chromatic aberration is obtained, as shown in the leftmost picture in the bottom row of Figure 4. Afterward, the R, G, and B channels are separated from the raw image, and images with low resolution are separated at the point where the focal plane is formed for each wavelength. By performing the FPM reconstruction algorithm based on the image array for the focal plane of each wavelength, it is finally possible to reconstruct a high-resolution image at three focal planes for each wavelength simultaneously. The FPM reconstruction algorithm is based on the principle that illuminating an object with tilted plane waves is equivalent to shifting the Fourier spectrum in the Fourier plane by the corresponding tilt angle [17]. The image array obtained with plane waves tilted at different angles is shifted in the Fourier plane according to each angle and then combined. By repeating this process multiple times, a high-resolution reconstructed image can be obtained.

**Figure 5:**
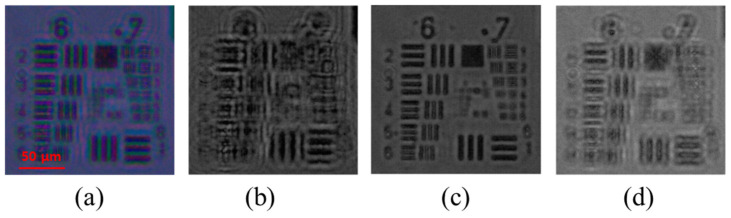
RGB channels split from the raw image captured using a color camera. (**a**) Image acquired with all RGB LEDs turned on, (**b**) R channel image, (**c**) G channel image, and (**d**) B channel image.

Figure 5a is an image obtained from the color camera with all RGB LEDs turned on. To accurately analyze the resolution of the captured image, the 1951 USAF resolution chart was placed at the focus point of the objective lens, and the image was captured. The 1951 USAF resolution chart was located at the focal point corresponding to a wavelength of 520 nm. Figure 5b–d shows the R, G, and B channels, respectively. Except for the image of the G channel where the focal plane is located, the R channel and B channel image qualities are deteriorated due to defocusing by chromatic aberrations [18].

## 3. Results

**Figure 6:**
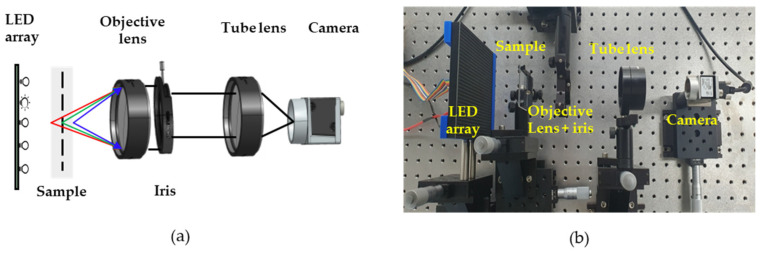
Simultaneous multifocal plane FPM. (**a**) Schematic diagram and (**b**) experimental setup.

Simultaneous multifocal plane FPM was experimentally demonstrated using the set-up shown in Figure 6. The designed NA of the aspherical lens was 0.2, but peripheral aberration occurred when full aperture was used, so the experiment was conducted by placing an iris and setting the NA of the aspherical lens to 0.15 and 9.6× magnification. Since the simultaneous multifocal plane FPM has the same configuration as the existing FPM system, it does not require additional hardware. The LED array light source consists of 1024 RGB LEDs arranged in a 32 × 32 grid with 4 mm grid spacing. The distance between the LED array and the measurement sample is 100 mm, and the gap between each LED in the array is 4 mm. The LEDs used in FPM reconstruction involved those in the 5 × 5 grid located in the center of the array. When an LED chip located farther from the center was used to further increase the NA of the illumination, image quality deteriorated due to reduced light intensity and lens aberration, which made the image unsuitable for use as raw data for the FPM reconstruction algorithm. Therefore, the NA of the illumination is 0.08, and the synthetic NA of the FPM system is the sum of the NA of the illumination and the NA of the objective lens, which has a synthetic NA of 0.23.

**Figure 7:**
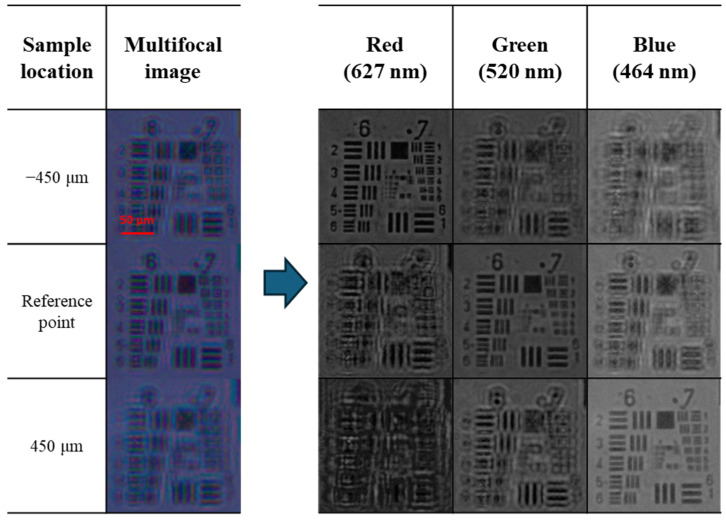
Channel-splitting images acquired at the focal planes for 627 nm (R), 520 nm (G), and 464 nm (B) wavelength illumination.

Figure 7 shows captured images with all the RGB LEDs located in the center turned on and the USAF 1951 target positioned at the focal plane corresponding to each wavelength. At the focal distance of the central wavelength of the LED array, 520 nm, there is a difference of −450 μm observed for the focal distance formed by light at 627 nm, while the focal distance formed by light at 464 nm is confirmed to be positioned at +450 μm.

**Figure 8:**
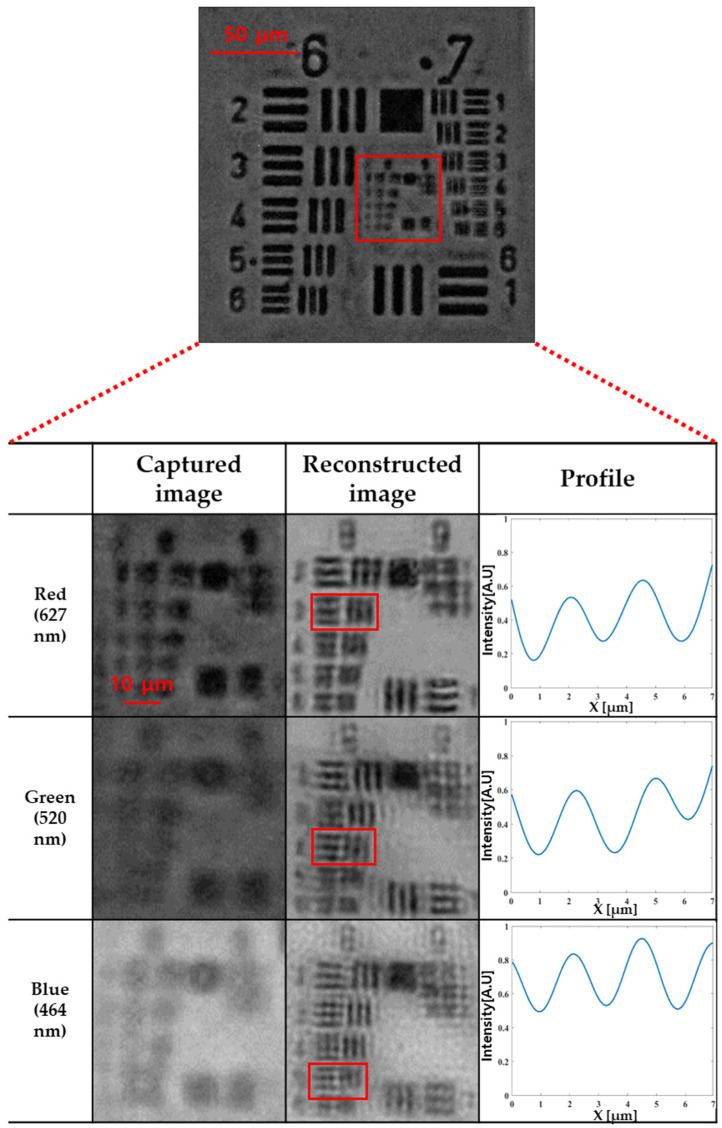
Reconstructed image of R, G, B image, 8 Group 3 element profile of red reconstructed image, 8 Group 4 element profile of green reconstructed image, and 8 Group 5 element profile of reconstructed image.

Figure 8 shows the reconstructed image of the R, G, and B channel image, 8 Group 3 element profile of the red reconstructed image, 8 Group 4 element profile of the green reconstructed image, and 8 Group 5 element profile of the blue reconstructed image. To evaluate the resolution of the FPM image, we calculated the contrast ratio using the image profile of the resolution chart. The contrast ratio is calculated as follows: contrast ratio = (I_max_ − I_min_)/(I_max_ + I_min_), where I_max_ and I_min_ are the maximum and minimum intensities of the image, respectively. Generally, in optical systems, if the contrast ratio is over 0.2, the spatial resolution is deemed acceptable. The resolution calculated at the 627 nm wavelength used in the R channel image and with a synthetic NA of FPM of 0.23 is 1.66 μm using the following formula: resolution = 0.61∙λ/NA. The measured contrast ratio of group 8 element 3 with a bar/space width of 1.55 μm is 0.32, as shown in Figure 8 reconstructed image of red, which can be determined to meet the contrast ratio criteria. The G-channel image has a resolution of 1.37 μm, which corresponds to element 4 of Group 8 of the USAF Resolution Test Chart. The measured contrast ratio of group 8 element 4 is 0.22, as shown in Figure 8 reconstructed image of green, which meets the contrast ratio criteria. The resolution of the B channel image is 1.23 μm, which roughly corresponds to element 5 of group 8 in the test chart. The measured contrast ratio of element 5 of group 8 is 0.25, as shown in Figure 8 reconstructed image of blue, which meets the contrast ratio criteria, confirming that the measured resolution is similar to the calculated value.

## 4. Transparent OLED Measurement Using Simultaneous Multifocal Plane FPM 

**Figure 9:**
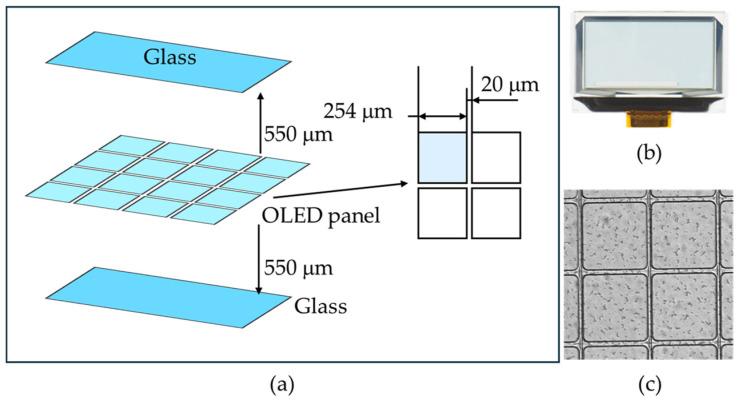
Transparent OLED used for evaluating the multifocal imaging performance. (**a**) Schematic structure of OLED. The OLED panel is protected by a glass substrate with a thickness of 550 μm above and below. The size of the OELD pixel is 254 μm, and the spacing between each OLED pixel is 20 μm. (**b**) Photo of the transparent graphical OLED. (**c**) The transparent OLED magnified and imaged with an objective lens with an NA of 0.13 and magnification of 4.

Simultaneous multifocal plane FPM can used to image various biological and industrial samples. Transparent organic light-emitting diodes (OLED) were inspected using the simultaneous multifocal plane FPM system developed in this study. The structure and shape of the transparent OLED (Qwiic Transparent Graphical OLED Breakout, SparkFun) are shown in Figure 9.

When inspecting transparent OLEDs, there may be defects in the OLED pixels and/or scratches in the glass substrate layer. Since the gap between the glass substrate and the OLED pixel layer is 550 μm, it is impossible to image both layers simultaneously even if the depth of focus is greatly expanded in an existing microscope and if a wide depth of focus is secured using a lens with a small NA, the resolution is poor. When multifocal plane FPM is used, two or more layers can be observed simultaneously within a single image capture without an additional hardware-based focusing process.

**Figure 10:**
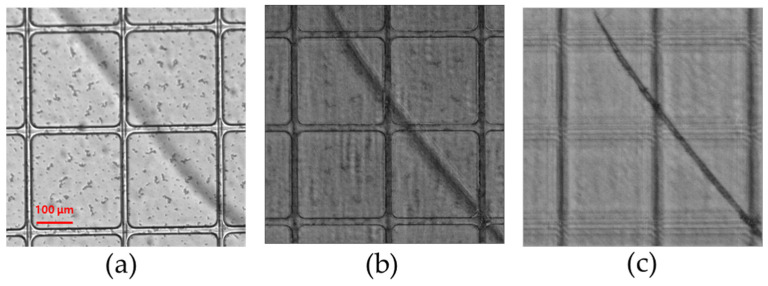
(**a**) Transparent OLED imaged using an objective lens with an NA of 0.13. (**b**) Reconstructed image using simultaneous multifocal plane FPM (R channel). (**c**) Reconstructed image using simultaneous multifocal plane FPM (G channel).

Figure 10a shows the OLED pixel layer in focus using an objective lens with an NA of 0.13. It is possible to observe one OLED pixel using conventional microscopy, but the scratches expected on the upper and lower glass substrate surfaces cannot be accurately imaged due to the limited DOF. Additionally, a separate focal length adjustment is required to determine which particular glass substrate is scratched. Figure 10b image is an image taken by focusing on one transparent OLED pixel using simultaneous multifocal plane FPM. After the FPM separation and reconstruction algorithms are performed, the OLED pixel is focused only on the R channel image. In addition, the G channel image reveals the scratches on the glass substrate, as shown in Figure 10c. Because the wavelength of the optical field used in the G channel image is longer than the wavelength used in the R channel image, the focal distance of the G channel is shorter, and it can resolve the scratches on the top glass substrate without requiring the sample to be moved along the z-axis.

## 5. Conclusions

FPM is a computational imaging technology that can reconstruct high-resolution images using low-resolution images with various Fourier information. In this study, we developed a simultaneous multifocal plane FPM system using an RGB LED array for illumination and verified its multifocal imaging performance. By turning on the RGB LEDs simultaneously, a raw image can be captured using a standard color camera. Then, the R, G, and B channels of the acquired image are separated to obtain multifocal plane images corresponding to the LED RGB wavelengths. Using the 1951 USAF resolution test chart, we verified that multifocal high-resolution images could be obtained by the FPM image reconstruction algorithm. Additionally, the strong potential of simultaneous multifocal plane FPM for industrial applications was demonstrated by using the developed system to inspect a transparent OLED device. Multifocal plane FPM was able to resolve and locate defects in the glass substrate and image the OLED pixel simultaneously with one image capture, significantly shortening the inspection time. 

In this work, we used a single commercially available aspherical lens with a low NA that is not optimized for the FPM system. Consequently, the resolution of the system can be enhanced if a multi-lens system with high NA is used. Moreover, more than three simultaneous focal planes may be possible using an image acquisition system that can handle different wavelengths. These strategies will be the focus of future research on simultaneous multifocal FPM.

## Figures and Tables

**Table 1:** Design parameters of the AL2550G-A aspheric lens.

Parameter		Value
Aspherical surface parameter	Y radius	25.9736
	Conic constant	−1.001
	4th—order coefficient	3.0408 × 10^−6^
	6th—order coefficient	7.2060 × 10^−10^
	8th—order coefficient	2.3292 × 10^−14^
Lens thickness		5.8 mm
Lens material		NBK-7 (Abbe number: 64.17, $n_{g}$: 1.5414)
Numerical aperture (NA)		0.2
Clear aperture		21.3 mm
Working distance		46.2 mm
Effective focal length		50.0 mm

**Table 2:** Specifications of the color camera used to acquire FPM images.

Parameters	Value
Sensor size	6.44 mm × 4.62 mm
Resolution	3040 pixels × 2748 pixels
Pixel size	1.67 μm × 1.67 μm
Mono/Color	Color

## Data Availability

The data presented in this study are available on request from the corresponding author. The data are not publicly available due to privacy.
